# Maskless micro/nanofabrication on GaAs surface by friction-induced selective etching

**DOI:** 10.1186/1556-276X-9-59

**Published:** 2014-02-04

**Authors:** Peng Tang, Bingjun Yu, Jian Guo, Chenfei Song, Linmao Qian

**Affiliations:** 1 Tribology Research Institute, National Traction Power Laboratory, Southwest Jiaotong University, Chengdu 610031, People’s Republic of China

**Keywords:** Maskless, Micro/nanofabrication, GaAs, Friction-induced selective etching

## Abstract

In the present study, a friction-induced selective etching method was developed to produce nanostructures on GaAs surface. Without any resist mask, the nanofabrication can be achieved by scratching and post-etching in sulfuric acid solution. The effects of the applied normal load and etching period on the formation of the nanostructure were studied. Results showed that the height of the nanostructure increased with the normal load or the etching period. XPS and Raman detection demonstrated that residual compressive stress and lattice densification were probably the main reason for selective etching, which eventually led to the protrusive nanostructures from the scratched area on the GaAs surface. Through a homemade multi-probe instrument, the capability of this fabrication method was demonstrated by producing various nanostructures on the GaAs surface, such as linear array, intersecting parallel, surface mesas, and special letters. In summary, the proposed method provided a straightforward and more maneuverable micro/nanofabrication method on the GaAs surface.

## Background

Due to its direct bandgap and high electron mobility, gallium arsenide (GaAs) has become one of the most widely used compound semiconductor materials. For instance, GaAs is the perfect substrate for quantum luminescent devices, such as photoelectric detector [1], high-performance laser [2], quantum information processing [3], and so on. Nevertheless, the precondition for realizing these quantum devices is to grow quantum dots on certain positions of substrate [4,5]. Thus, the controllable fabrication of the patterned GaAs substrate is a significant issue of concern.

Many efforts have been made in producing patterned GaAs substrate. As a prevalent technology, the photolithography has been used for the fabrication on GaAs surface [6]. However, the conventional photolithography has the embarrassment in its resolution vs. cost, i.e., the production cost will be much higher as the resolution is improved [7]. Although the electron beam (EB) [8] and focused ion beam (FIB) [9] lithography technology can enable higher machining precision and finer resolution patterning, these techniques are costly and complex, requiring multiple-step processes [10]. In addition, anodic oxidation has been demonstrated the potential in the creation of structures, patterns, and devices at the nanometer scale [11], but the operation will be controlled under rigorous environmental requirements, such as applied voltage, suitable humidity, and so on [12]. Such processes can also bring contamination and impurity onto the area fabricated [13]. In recent decades, the proximal probe method based on the mechanical stamp and scratching technique has been employed to produce patterned GaAs substrate [4,14], but it is difficult, if not impossible, to fabricate GaAs nanostructures with low destruction by solely mechanical scratching. Therefore, it is necessary to develop a straightforward and more flexible fabrication method for the GaAs surface.

In the present study, a novel friction-induced micro/nanofabrication method that consists of nanoscratching and post-etching was presented to produce nanostructures on GaAs. The effects of the applied normal load and etching period on the formation of the nanostructure were studied. Based on the X-ray photoelectron spectroscope (XPS) and Raman spectra characterization, the fabrication mechanism of the nanostructure was discussed. Finally, through a homemade multi-probe instrument, the capability of this fabrication method was demonstrated by producing various nanostructures on the GaAs surface, such as linear array, intersecting parallel, surface mesas, and special letters.

## Methods

### Material

The GaAs (100) wafers, n-doped with Si, were purchased from JMEM Electronic Materials, Ltd., Tianjin, China. Using an atomic force microscope (AFM, SPI3800N, Seiko, Tokyo, Japan), the surface root-mean-square (RMS) roughness of the GaAs wafer was measured as 0.5 nm over a 1 μm × 1 μm area. The crystal state of the GaAs material was detected by the X-ray diffraction (XRD, X'Pert, PANalytical, Almelo, Netherlands), showing that the GaAs wafer was single crystal in (100) plane orientation. Before the fabrication, the GaAs wafers were ultrasonically cleaned with methanol and ethanol for 3 min in turn, and successively rinsed with deionized water for 10 min to remove surface contamination.

### Fabrication method

**Figure 1:**
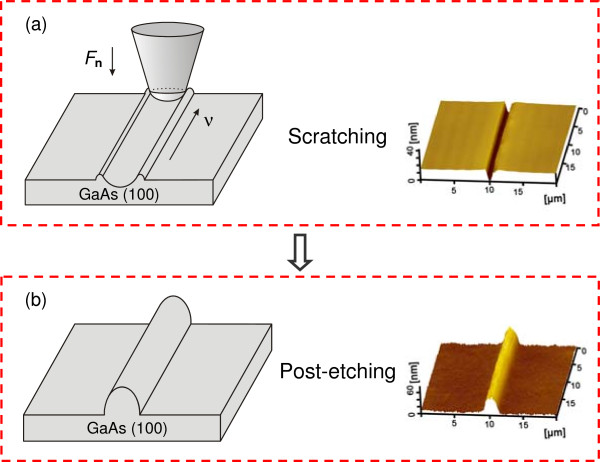
**Schematic illustration showing the friction-induced selective etching on GaAs surface. (a)** A groove was formed on GaAs surface after scratching a diamond tip under a normal load of *F*_n_. **(b)** A protrusive nanoline was created on GaAs surface after post-etching in H_2_SO_4_ aqueous solution.

As shown in Figure 1, the maskless fabrication process consists of scratching and post-etching. When the GaAs surface was scratched by a diamond tip along the designed traces, grooves can be generated on the scanned area. After etching in H_2_SO_4_ aqueous solution, different protrusive nanostructures can be produced *in situ* from the scratched area on the GaAs surface. Scratching tests on the GaAs surface were performed by a nanoscratch tester (CSM Instruments, Peseux, Switzerland) or a homemade multi-probe instrument [15]. The spherical diamond tips used for scratching have the radii of about 5 μm. After the scratching tests, the specimens were dipped in a mixture of H_2_SO_4_ aqueous solution (H_2_SO_4_/H_2_O_2_/H_2_O = 1:0.5:100) for post-etching [16]. During scratching and post-etching, the experimental temperature was controlled at 22°C and the relative humidity varied between 50% and 55%. All the AFM images of GaAs specimens were scanned by silicon nitride tips (MLCT, Veeco Instruments Inc., Plainview, NY, USA) with the spring constant *k* = 0.1 N/m in vacuum. The morphology of GaAs surface patterns was observed by a scanning electron microscope (SEM, QUANTA200, FEI, Hillsboro, OR, USA).

### XPS and Raman characterization

In order to investigate the mechanism of the friction-induced selective etching process, the mesas with an area of 500 μm × 500 μm and a height of 60 nm were prepared by the homemade multi-probe instrument under a normal load of 10 mN and post-etching for 30 min. The chemical state of the fabrication area on the GaAs surface was detected by an XPS (Thermo VG250, Thermo, Waltham, MA, USA). The microstructure of the fabrication area on the GaAs surface was measured using a Raman spectrometer (RM2000, Renishaw, Gloucestershire, UK). The excitation was supplied by the 514.5 nm Ar^+^ ion laser. To avoid the random error in detection, each sample was scanned for three times.

## Results and discussion

### Fabrication of GaAs nanostructures

#### ***Effect of etching period on friction-induced selective etching***

**Figure 2:**
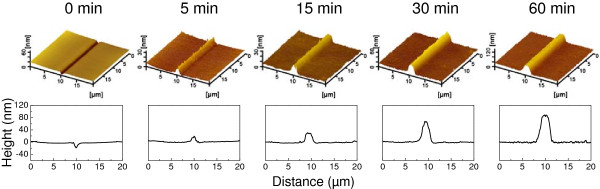
**Effect of etching period on fabrication of GaAs surface by scratching and post-etching.** The AFM images (top) and cross-sectional profiles (bottom) of the nanostructures were obtained after scratching under a normal load of 20 mN and post-etching in the H_2_SO_4_ aqueous solution for 5, 15, 30, and 60 min, respectively.

**Figure 3:**
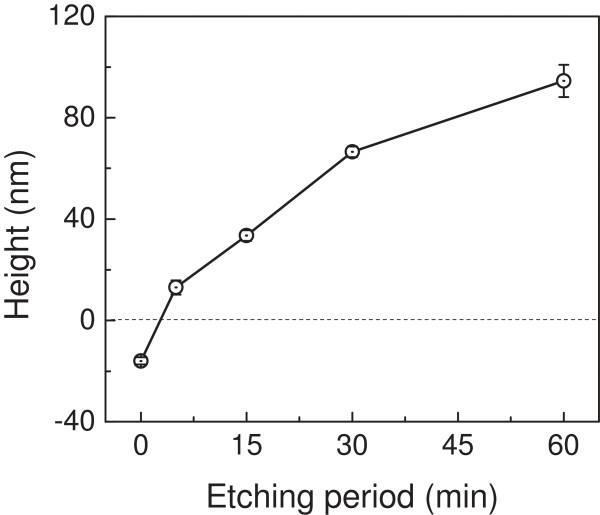
Effect of etching period on the height of the nanostructure on GaAs surface.

The etching period was found to show an obvious effect on the fabrication of GaAs nanostructures. After scratching on the GaAs surface under a normal load *F*_*n*_ of 20 mN, a groove with a depth of about 15 nm was created on the GaAs surface. Subsequently, a protrusive nanostructure was observed on the groove area after dipping the specimen into H_2_SO_4_ aqueous solution for 5 min. Figure 2 showed the AFM images and cross-sectional profile curves of the protrusive nanostructures after scratching and post-etching. The variation of the height of these protuberances with etching period was plotted in Figure 3. It was observed that the height of GaAs protrusive structure gradually increased from 12 to 94 nm with the increase in etching period from 5 to 60 min. Such results indicated that the etching rate of the scratched area was much less than that of monocrystalline GaAs. The scratched area can act as an etching mask in H_2_SO_4_ solution.

#### ***Effect of normal load on friction-induced selective etching***

**Figure 4:**
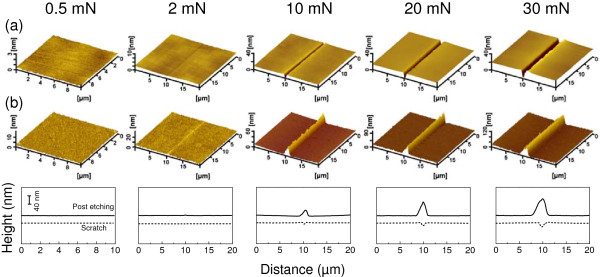
**Effect of normal load on the fabrication of GaAs surface by scratching and post-etching. (a)** AFM images of the scratches created on the GaAs surface under various normal loads. **(b)** AFM images of the nanolines on the GaAs surface after etching in H_2_SO_4_ aqueous solution for 30 min. The cross-sectional profiles were plotted below for the comparison.

**Figure 5:**
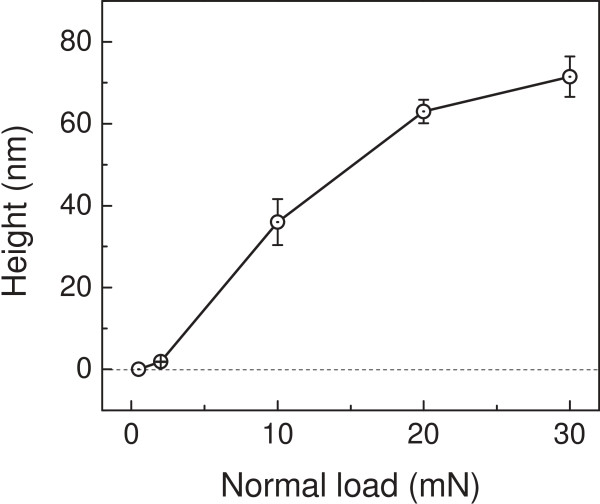
Effect of normal load on the height of the nanostructure on the GaAs surface.

Aside from the etching period, the normal load also reveals an effect on the fabrication of the GaAs surface. As shown in Figure 4a, scratching tests were performed on the GaAs surface under various normal loads ranging from 0.5 to 30 mN. When the normal load was 0.5 mN, there was no obvious scratching trace on the surface. When the normal load was increased to 2 mN, a slight groove with a depth of about 0.5 nm was formed on the GaAs surface. However, when the normal load exceeded 10 mN, the scratching damage became severe and the depth of the groove increased to 23 nm at 30 mN. After etching in H_2_SO_4_ aqueous solution for 30 min, there was no visible etching difference on the wearless scratched surface, as shown in Figure 4b. However, the protuberance piled up gradually from the groove area when the normal load increased from 2 to 30 mN. Therefore, the critical load for the friction-induced fabrication on the GaAs surface is 2 mN, under which the Hertzian contact pressure *P*_c_ is estimated as 4.85 GPa [17,18]. Such contact pressure was very close to the critical Hertzian contact pressure for the initial yield of GaAs surface [19]. The height of those protuberances was plotted in Figure 5. It can be seen that the height of these protuberances increased with the normal load during scratching. When the load was 30 mN, the height of nanostructures could get to 75 nm. Since the protuberance formed only in the wear area, the fabrication mechanism could be related to the deformation of the substrate induced by the mechanical interaction. The detailed generation mechanism of the protrusive nanostructures on the GaAs surface will be discussed in the next section.

### Mechanism of the friction-induced selective etching on GaAs surface

#### ***Effect of surface oxide on the friction-induced selective etching***

**Figure 6:**
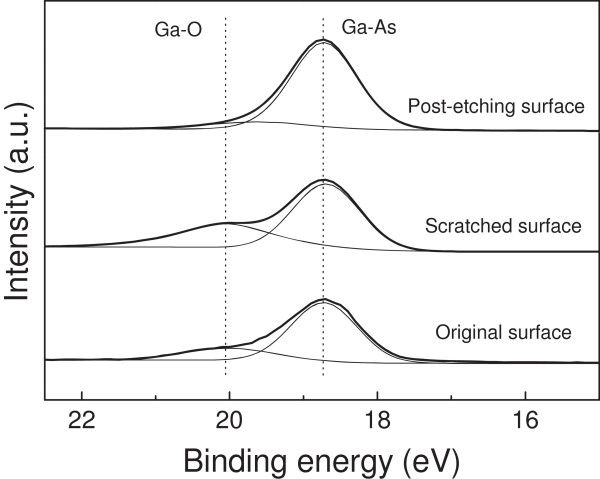
**XPS analysis on chemical bonding states of Ga element.** The detection were performed on original surface, scratched surface, and post-etching surface (scratched surface after etching) of GaAs, respectively.

Extensive work has shown that various nanostructures can be produced on monocrystalline silicon and quartz surfaces by the friction-induced selective etching method [20,21]. Guo et al. [22] suggested that both the tribochemical reaction and the transmutation of crystal structure on the scanned area can result in friction-induced selective etching. To investigate whether the tribochemical reaction played the role in the selective etching of the GaAs surface, X-ray photoelectron spectroscope was used to detect the possible change of chemical composition on the original surface, scratched surface, and post-etching surface, respectively. The variation in the bonding states of Ga was presented in Figure 6. On the original surface, it was observed that there were two Ga3*d* peaks, i.e., Ga-O (Ga_2_O_3_) bond at 20.05 eV and Ga-As bond at 18.74 eV [23], which meant that a native oxide layer existed on the sample surface. On the scratched area, the signal of Ga-O was a little stronger than that on the original surface. After etching for 30 min, the mask effect still existed and the signal of Ga-O was very weak. Such little oxide may come from the natural oxidation of GaAs surface during the period between the finish of sample preparation and the start of XPS detection. In addition, from the X-ray full spectrum of GaAs before and after scratching, no other element or chemical compound was found in the process of the fabrication beside GaAs and its oxide. All these results confirmed that only slight tribochemical oxidation occurred on the GaAs surface during scratching. Since it was reported that the oxide of GaAs has a higher solubility into H_2_SO_4_ solution than GaAs substrate [24], the oxide layer may not play a role as etching mask. Therefore, the scratch-induced structural deformation was expected to act as a mask during the generation of GaAs nanostructures in H_2_SO_4_ solution.

#### ***Effect of structural deformation on the friction-induced selective etching***

**Figure 7:**
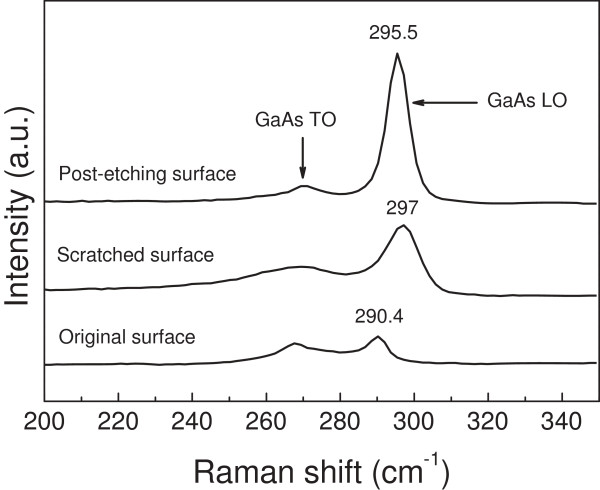
**Raman detection on GaAs surface.** The spectra were obtained from original surface, scratched surface, and post-etching surface (scratched surface after etching), respectively.

**Figure 8:**
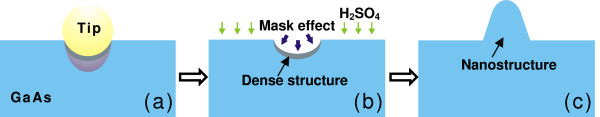
Schematic picture showing fabrication mechanism of GaAs nanostructure.

To verify whether the scratch-induced structural deformation occurred during the fabrication process, the Raman detection was conducted on original GaAs surface, scratched surface and post-etching surface. As shown in Figure 7, the Raman spectra of the original GaAs (100) displays both a longitudinal optical (LO) phonon at 290.4 cm^-1^ and transversal optical (TO) phonon at 267 cm^-1^[25,26]. After scratching, the LO Raman peak became wider and the positive frequency shift was 7 cm^-1^ compared to that on the original surface. When the post-etching was finished, the LO Raman peak of the mesa surface showed a negative shift of about 2 cm^-1^. The shift and broadening of the peaks can be ascribed to the structure disorder of GaAs lattice [27]. Moreover, the positive frequency shift of LO phase is a typical character of residual compressive stress. The higher the residual compressive stress, the greater the density of crystal structure [28,29]. As shown in Figure 8, the dense structure was expected to delay the diffusion of the etchant into internal GaAs substrate, which reduced the etching rate of the scratched area. Therefore, the dense structure can act as a ‘mask’ in the friction-induced selective etching of GaAs. It should be noted that compared to solely mechanical scratching, the GaAs nanostructures produced by the proposed method will have relatively lower destruction.

### Fabrication of surface pattern on GaAs surface

**Figure 9:**
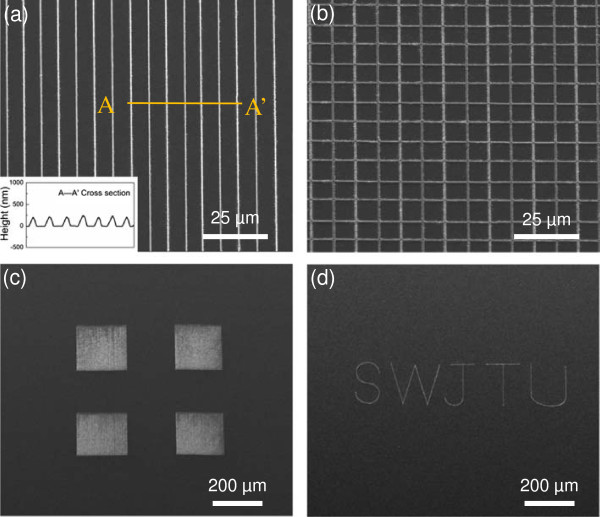
**SEM images of GaAs patterns fabricated by friction-induced selective etching. (a)** Linear arrays, **(b)** intersecting parallels, **(c)** surface mesas, **(d)** nanoletters ‘SWJTU’.

Based on the friction-induced selective etching method, different patterns were produced on the GaAs surface by a homemade multi-probe instrument [15]. Figure 9a displayed the linear array with the height of 200 nm and width of 2 μm. The lower left inset in Figure 9a showed the cross-sectional profile of the selected nanolines (marked by line A-A’). In Figure 9b, when the scanning traces were conducted, both on horizontal and vertical directions, intersecting parallels GaAs pattern were produced after post-etching for 2 h. The height of the GaAs nanolines was about 200 nm and the pitch width was about 9 μm. Such pattern may shed new light in orderly formation of the quantum dots or liquid drop in the manufacture process of quantum devices [30]. Figure 9c showed a 200 μm × 200 μm mesa array through continuous scanning at a normal load of 10 mN and post-etching for 1 h. In Figure 9d, the letters ‘SWJTU’ (short for Southwest Jiaotong University) on GaAs surface was ‘written’ by the scanning program control. Therefore, various patterned GaAs substrates can be achieved by controlling the normal load, scanning trace, and etching period on the GaAs surface. It is suited for large scale machining with more flexibility.

In summary, the present study proposed a friction-induced selective etching method on GaAs surface. XPS and Raman detection demonstrated that the residual compressive stress and the lattice densification was the main reason for the selective etching. Various patterns can be created on a target GaAs surface. Without any resist mask and applied voltages, this method provides a straightforward and more maneuverable micro/nanofabrication method on the GaAs surface.

## Conclusions

A friction-induced selective etching method was presented to fabricate nanostructures on GaAs surface. The effects of normal load and etching period on the formation of nanostructures were investigated. The mechanism for the selective etching was discussed based on the XPS and Raman analysis. The main conclusions can be summarized as below:

(1) Nanostructures can be created on the GaAs surface after scratching and post-etching in H_2_SO_4_ solution. The height of the nanostructures increased gradually with the increase in applied normal load or etching period.

(2) Based on the XPS and Raman detection, it was found that the residual compressive stress and lattice densification induced by the scratching process were probably the main reason for the friction-induced selective etching.

(3) Various nanostructures including line arrays and nanopatterns can be produced on the GaAs surface by the controlment of normal load, scanning trace, and etching period. Without any resist mask and applied voltages, the proposed method will open new opportunity for the micro/nanofabrication of GaAs.

## Abbreviations

AFM: atomic force microscope; EB: electron beam; FIB: focused ion beam; GaAs: gallium arsenide; LO: longitudinal optical; RMS: root-mean-square; TO: transversal optical; XPS: X-ray photoelectron spectroscope; XRD: X-ray diffraction.

## Competing interests

The authors declare that they have no competing interests.

## Authors’ contributions

PT, JG, and CS finished the fabrication experiments and acquired the original data in this article. LQ and BY have made substantial contributions to the conception and design for this article. All the authors read and approved the manuscript.
